# Novel ORAI1 Mutation Disrupts Channel Trafficking Resulting in Combined Immunodeficiency

**DOI:** 10.1007/s10875-021-01004-8

**Published:** 2021-03-01

**Authors:** Fang Yu, Nourhen Agrebi, Rafah Mackeh, Khaled Abouhazima, Khadija KhudaBakhsh, Mehdi Adeli, Bernice Lo, Amel Hassan, Khaled Machaca

**Affiliations:** 1 Department of Physiology and Biophysics, Weill Cornell Medicine Qatar, Education City, Qatar Foundation, Doha, Qatar; 2 Calcium Signaling Group, Weill Cornell Medicine Qatar, Education City, Qatar Foundation, Doha, Qatar; 3 Translational Medicine Department, Sidra Medicine, Doha, Qatar; 4 Pediatric Gastroenterology, Sidra Medicine, Education City, Doha, Qatar; 5 General Pediatrics, Sidra Medicine, Education City, Doha, Qatar; 6 Pediatric Allergy and Immunology Department, Sidra Medicine, Education City, Doha, Qatar; 7 College of Health and Life Sciences, Hamad Bin Khalifa University, Doha, Qatar

**Keywords:** Combined immunodeficiency, ORAI1, store-operated Ca^2+^ entry, trafficking, integral membrane protein, channel, Ca^2+^ signaling, immune cell function, myotonia, anhidrosis

## Abstract

Store-operated Ca^2+^ entry (SOCE) represents a predominant Ca^2+^ influx pathway in non-excitable cells. SOCE is required for immune cell activation and is mediated by the plasma membrane (PM) channel ORAI1 and the endoplasmic reticulum (ER) Ca^2+^ sensor STIM1. Mutations in the *Orai1* or *STIM1* genes abolish SOCE leading to combined immunodeficiency (CID), muscular hypotonia, and anhidrotic ectodermal dysplasia. Here, we identify a novel autosomal recessive mutation in ORAI1 in a child with CID. The patient is homozygous for p.C126R mutation in the second transmembrane domain (TM2) of ORAI1, a region with no previous loss-of-function mutations. SOCE is suppressed in the patient’s lymphocytes, which is associated with impaired T cell proliferation and cytokine production. Functional analyses demonstrate that the p.C126R mutation does not alter protein expression but disrupts ORAI1 trafficking. Orai1-C126R does not insert properly into the bilayer resulting in ER retention. Insertion of an Arg on the opposite face of TM2 (L135R) also results in defective folding and trafficking. We conclude that positive side chains within ORAI1 TM2 are not tolerated and result in misfolding, defective bilayer insertion, and channel trafficking thus abolishing SOCE and resulting in CID.

**Supplementary Information:**

The online version contains supplementary material available at 10.1007/s10875-021-01004-8.

## Introduction

Store-operated Ca^2+^ entry (SOCE) is ubiquitous Ca^2+^ influx pathway that regulates cellular signaling [1–4]. SOCE is triggered downstream of PLC-linked agonists that result in the production of IP_3_ and Ca^2+^ release from stores. Intracellular Ca^2+^ stores depletion is sensed by the resident ER transmembrane protein STIM1, which clusters and migrates to ER-PM contact sites (ER-PM CS) that are in close apposition to the PM (within 25–30 nm) [1–3]. STIM1 recruits ORAI1 through diffusional trapping and gates it open to trigger Ca^2+^ influx [1–3].

Gain-of-function (GoF) and loss-of-function (LoF) mutations in either ORAI1 or STIM1 in humans lead to distinct pathologies [3, 5–7]. Autosomal dominant GoF mutations in ORAI1 that result in excessive Ca^2+^ influx including p.S97C, p.G98S, p.L138F, and p.P245L, develop TAM/Stormorken syndrome with no obvious immune phenotype [3, 8–10]. By contrast, recessive LoF mutations that abolish SOCE, including p.A88SfsX25, p.R91W, p.G98R, p.A103E/p.L194P (compound het.), p.H165PfsX1, p.V181SfsX8, and p.L194P, result in combined immunodeficiency (CID), anhidrotic ectodermal dysplasia (AED), and muscular hypotonia [3, 5–7, 11–15]. As the SOCE channel in lymphocytes is referred to as the Ca^2+^-release activated Ca^2+^ channel (CRAC), these pathologies are known as CRAC-channelopathies.

ORAI1 is composed of four transmembrane domains (TM1–TM4) with cytoplasmic N- and C-termini. The mature CRAC channel is a hexamer, in which six TM1 domains define the channel pore, and are surrounded by interlocking TM2 and TM3 helices and peripheral TM4 domains [16, 17].

Here we report the identification of a patient with a novel autosomal recessive mutation in ORAI1 associated with typical CRAC channelopathies, including CID, muscular hypotonia, and anhidrosis. The C126R mutation in ORAI1 TM2 abolishes SOCE in patient PBMCs and does not support SOCE when expressed in ORAI1-KO cells. Functional analyses reveal that C126R is unable to traffic to the PM and is rather retained in the ER. This is due to the positively charged Arg side chain in TM2, since introduction of a positive charge on the opposite face of TM2 (L135R) results in the trapping of ORAI1 in the ER, whereas a polar side chain substitution (C126S) has no effect on ORAI1 trafficking.

## Methods

### Patients

Sample collection was performed with informed consent from the patient family (of Indian descent) according to the declaration of Helsinki, and experimentation was performed following institutional IRB approval. The peripheral blood mononuclear cells (PBMCs) were prepared by the Ficoll-Paque plus (GE Healthcare) density gradient separation method following the manufacturer’s protocol.

### In Vitro T Cell Proliferation Assay and T Cell Populations

0.5–1.5 × 10^6^ PBMCs were washed twice in PBS then stained with 1.25 μM of carboxyfluorescein succinimidyl ester (CFSE) for 15 min at 37 °C in dark. After five washes in complete medium, cells were incubated with anti-CD2, anti-CD3, and anti-CD28 antibodies from the T cell activation/expansion kit (Miltenyi Biotec) according to the manufacturer’s instructions. At day 3, cells were stained for surface markers (day 3 cells) or cultured for 2 more days in the presence of 100 U/mL of IL-2 (day 5 cells). Staining for CD4 and CD8 and viability dye was carried out for 30 min on ice, using V450 anti-CD4 and BV605 anti-CD8 (BD Biosciences) along with viability dye (LIVE/DEAD™ Fixable Near-IR Dead Cell Stain Kit, ThermoFisher). For Tregs identification, cells were incubated with antibodies against surface markers (anti-CD25-PE, CD3-FITC, CD127-AF647) for 45 min on ice, washed, fixed/permeabilized, and incubated with CD4-V450 and Foxp3-PE-cy7 (eBioscience). Cells were acquired on the NovoCyte flow cytometer and analyzed using FlowJo v10.

### Analysis of Cytokine Production

Activated T cells were re-stimulated with 20 nM phorbol 12-myristate 13-acetate (PMA) (Sigma-Aldrich) and 1 μM ionomycin (Sigma-Aldrich). After incubation for 1 h at 37 °C, a protein transport inhibitor Brefeldin-A (Invitrogen) was added, followed by 4-h incubation. Surface Fc receptors were blocked for 15 min at 4 °C using Human TruStain FcX™ (Biolegend). Cells were stained with anti-CD8-APC/Fire™750, fixed with BD Cytofix/Cytoperm (BD Biosciences) and stained with anti-CD4-AF488 (BD Biosciences), anti-IFN-γ-PE (BD Biosciences), anti-TNF-α-BV650 (BD Biosciences), and IL-2-APC (eBioscience). Cells were acquired on the NovoCyte flow cytometer and analyzed using FlowJO v10.

### DNA Sequencing and Segregation Analysis

Genomic DNA was isolated from blood using the DNeasy blood and tissue kit (Qiagen) according to the manufacturer’s instructions. PCR was performed using the following primers: 5′-gaaaactgaggctcggagag-3′ and 5′-AGCACCACCTCAGCTAGGAA-3′. PCR products were Sanger sequenced and analyzed using Unipro UGENE.

### Real-Time Quantitative PCR

RNA was extracted from 5 × 10^6^ activated T cells using RNeasy kit (Qiagen). cDNA was synthesized from mRNA using oligo(dT) primer and MML-V reverse transcriptase (Invitrogen) according to the manufacturer’s instructions. The ORAI1 transcript was quantified using Fast SYBR Green Master Mix (Applied Biosciences) using the following primers: forward 5′-CCATGGTGGCAATGGTGGAGG-3′; reverse 5′- GTTGGGCAGGATGCAGGTGC -3′. All reactions were performed in triplicate in a QuantStudio 6 K Flex real-time PCR machine (Applied Biosciences). Data were normalized to the expression of the house-keeping gene *RPLP0* (Ribosomal protein, large, P0) by calculating 2^-ΔCT^, with ΔCT the difference in CT values for ORAI1 transcript and RPLP0 gene.

### Primers for Mutagenesis

The primer pairs used for mutagenesis are listed below:

- C126R-forward 5′-CATCGCCTTCAGTGCCCGCACCACAGTGCTGGTGGCTG-3′
- C126R-reverse 5′-CAGCCACCAGCACTGTGGTGCGGGCACTGAAGGCGATG-3′
- L135R-forward 5′-CTGGTGGCTGTGCACCGGTTTGCGCTCATGATC-3′
- L135R-reverse 5′-GATCATGAGCGCAAACCGGTGCACAGCCACCAG-3′
- C126S-forward 5′-CATCGCCTTCAGTGCCAGCACCACAGTGCTGGTGGCTG-3′
- C126S-reverse 5′-CAGCCACCAGCACTGTGGTGCTGGCACTGAAGGCGATG-3′

The double mutation pDS_YFP-HA-Orai1-C126R/L135R was generated based on single mutation. All mutations used in experiments were sequencing confirmed.

### Cell Culture and Transfection

To activate and expand T cells from PBMCs, thawed PBMCs from patient, patient’s father, or healthy donor resuspended in 10% complete RPMI 1640 medium [18] were stimulated with 1 μg/ml of anti-CD3 (clone HIT3a, BD Pharmingen 555336) and anti-CD28 (clone CD28.2, BD Pharmingen 555725) antibodies, and plated at 2 × 10^6^ cells per well of 48-well plate. PMBCs were incubated at 37 °C and under 5% CO_2_ for overnight and then continue cultured in the presence of 30 U/ml hIL-2 (PeproTech) in medium for 7 days. Fresh hIL-2 containing medium was added every 2 days. Orai1-KO HEK293 [19] cells were in DMEM media (Invitrogen) supplemented with 10% FBS (Gibco), 100 U/ml penicillin, and 100 μg/ml streptomycin. Transient transfections were performed using Lipofectamine 2000 (Invitrogen) according to the manufacturer’s protocol. The transfection efficiency was routinely checked by low magnification microscopy on an EVOS FL Cell Imaging System (ThermoFisher Scientific). For 4-PBA treatment, cells were first transfected with plasmids for 6 h and then treated with 10 μM of 4-PBA for additional 12 h.

### Western Blots

PBMC lysates were subjected to SDS-PAGE using NuPAGE 4–12% Bis-Tris Gels (Invitrogen). The procedure was described previously [20]. Primary antibodies used are Orai1 polyclonal antibody (ProSci #4041) and α-Tubulin monoclonal antibody (Cell Signaling Technology #2144). Both HRP-conjugated goat anti-rabbit and anti-mouse IgG antibodies were obtained from Jackson ImmunoResearch Laboratories.

### Plasmids

Plasmids pDS_GFP-myc-ORAI1 [21] and pDS_YFP-HA-ORAI1 [22] were a gift from Rich Lewis (Stanford), and the pcDNA3.1(+)_mCherry-ORAI1 plasmid was described previously [20]. To construct human ORAI1-C126R and ORAI1-L135R mutants, pDS_GFP-myc-ORAI1, pDS_YFP-HA-ORAI1 and pcDNA3.1(+)_mCherry-ORAI1 plasmids were used as templates for site directed mutagenesis of C126 and L135 for R substitution using the Quickchange mutagenesis kit (Agilent Technologies).

### Confocal Microscopy

ER-Tracker green was purchased from Invitrogen. Cells were cultured on the 35-mm glass-bottom dishes (MatTek) at 37 °C with 5% CO_2_ and imaged in Ringer solution containing (in mM) 155 NaCl, 4.5 KCl, 2 CaCl_2_, 1 MgCl_2_, 10 D-glucose, and 5 Na-HEPES, pH 7.4. Imaging was performed on a Zeiss LSM 880 confocal with Airyscan using a Plan Apo 63×/1.4 oil DIC II objective with the pinhole at 1 AU. Images were analyzed using Imaris package (Bitplane) and figures compiled using Adobe Photoshop and Illustrator.

### Fluorescence Protease Protection (FPP) Assay

The FPP assay was performed as previously described [23]. Briefly, HEK293 cells transiently transfected with either pDS_GFP-myc-ORAI1 or pDS_GFP-myc-ORAI1-C126R plasmids were plated onto 35-mm glass-bottom dishes (MatTek). Cells were washed and replaced with KHM buffer (110 mM potassium acetate, 20 mM HEPES pH 7.4, and 2 mM MgCl_2_) at room temperature at 24 h after transfection. Epifluorescence images were taken before and 1 min after treatment with 20 mM digitonin and 50 μg/ml proteinase K.

### Ca^2+^ Imaging

Thapsigargin and Fura 2-AM were purchased from Invitrogen. Equal numbers of cells were stained with Fura2-AM for 30 min at 37 °C, washed with PBS, and transferred into the glass bottom 96-well plate (Corning Costar #3603). The plate was centrifuged at 3000 rpm for 3 min at room temperature. Ca^2+^ imaging was performed on a FlexStation 3 Multi-Mode Microplate Reader (Molecular Devices). SOCE levels were calculated by subtracting the basal 340/380 values before Ca^2+^ addition from the highest value after Ca^2+^ add-back.

### Statistics

Data are presented as mean ± SEM. Groups were compared using the Prism 8 software (GraphPad) using a one-way ANOVA. Statistical significance is indicated by the p value as ***p < 0.0001.

## Results

### Clinical Findings

**Fig. 1:**
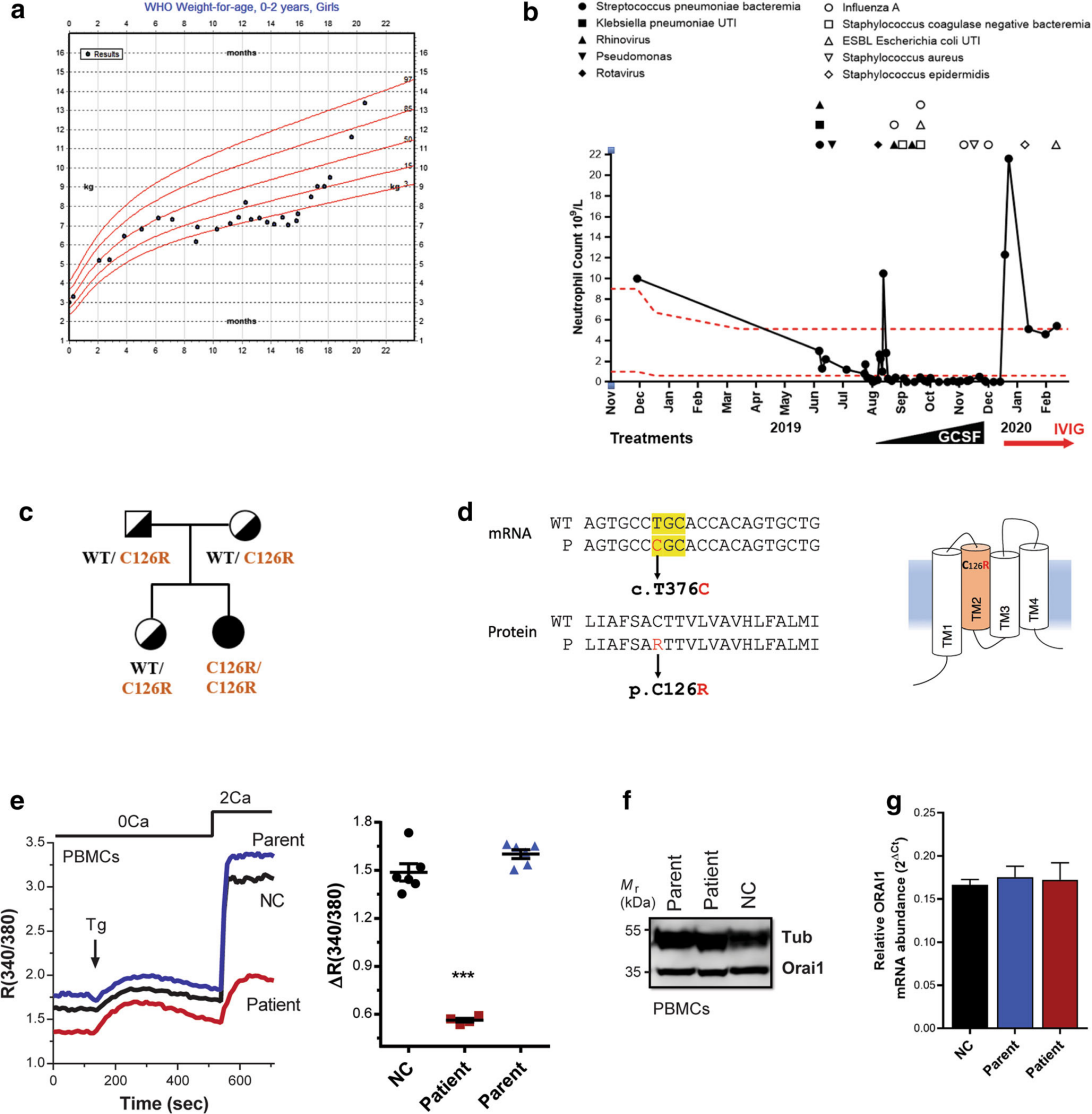
A novel *ORAI1* mutation associated with CRAC channelopathy. **a** Profile of patient body weight with World Health Organization (WHO) weight-for-age percentiles for girls (birth to 24 months) denoted in red. Body weight measurements are indicated as dots. **b** Neutrophil counts plotted over time. Infections and treatments are indicated above and below the graph respectively. Red dotted lines denote the reference range for neutrophil counts. UTI, urinary tract infection; GMCSF, granulocyte-macrophage colony-stimulating factor; IVIG, intravenous immunoglobulin. **c** Pedigree of the kindred. **d** mRNA and protein sequences of the *ORAI1* mutation identified in the patient. **e** Representative traces and summary data of SOCE responses from PBMCs from patient, parent, or healthy donor. PBMCs were incubated in Ca^2+^-free media and treated with the ER ATPase blocker thapsigargin (Tg) to deplete Ca^2+^ stores. This is followed by perfusion with Ca^2+^-containing medium (2 mM, 2Ca) to stimulate and quantify SOCE (*n* = 4 measurements, mean ± SEM; *p* < 0.0001, one-way ANOVA). **f** Western blot to assess ORAI1 protein levels in PMBCs from patients, patient’s father (parent), and healthy donor (NC). α-Tubulin was used as a loading control. **g** *ORAI1* mRNA expression in the patient, normal control (NC), and a parent. ORAI1 mRNA expression was normalized to house-keeping ribosomal protein RPLP0 expression and shows no difference between the patient, parent, or healthy donor. Mean + SEM, *n* = 3 technical replicates

A 22-month old female patient presented with a combination of severe infections, muscular hypotonia, and anhidrosis in the first year of life (clinical features summarized in Table 1). At 7 months of age, the patient was not able to achieve her motor milestones, consistent with congenital muscular hypotonia. The patient developed normally until the age of 5 months (Fig. 1a), but was admitted to the pediatric intensive care unit and ventilated at 9 months of age as she suffered from frequent recurrent severe bacterial and viral infections, which caused bronchopneumonia, bacteremia, and gastroenteritis (Fig. 1b). The patient continued to be hospitalized for 6 months, with persistent fever, chronic cough, and respiratory distress needing at least 6 l of oxygen. After 9 months of age, the patient developed severe persistent neutropenia, which was unresponsive to GCSF and has further contributed to frequent infections (Fig. 1b). Anti-neutrophil antibodies were negative, but the neutropenia responded well to an intravenous immune modulatory dose of immunoglobulin (Fig. 1b, IVIG). The patient had gastrointestinal complications such as chronic diarrhea, which was sometimes bloody, and abdominal distention with a picture of frank colitis, resulting in failure to thrive (Fig. 1a). The patient was initially gaining weight from birth till 6 months of age but started to lose weight after hospital admission and failed to thrive until supported with total parenteral nutrition (Fig. 1a). In addition, the patient failed the sweat test three times because of anhidrosis and had abnormal teeth with very thin enamel and irregular surface. Her hair was normal with no concerns, but she did have a recurrent erythematous rash that responded only partially to cortisone alone and resolved after applying cortisone and topical antibiotics. Differential lymphocyte counts show normal levels of CD8^+^ T cells, CD19^+^ B cells, and NK cells, with slightly increased levels of T_h_ cell (CD4^+^) (Fig. S1A). Furthermore, there was pronounced increase in the levels of IgG and IgA in the patient (Fig. S1A).

**Table 1:** Summary of clinical findings in the patient

	Findings
Inheritance	Autosomal recessive
Type of mutation	Missence (c. T376C)
Orai1 mutation	p.C126R within transmembrane domain 2 of ORAI1
Orai1 expression	Normal mRNA and protein levels. ORAI1 PM localization abolished.
SOCE	SOCE abolished in PMBCs
Infections	*Streptococcus pneumoniae* bacteremia; *Klebsiella pneumoniae* urinary tract infection; Rhinovirus (viral bronchopneumonia); perianal abscess (Pseudomonas from wound culture); Rotavirus gastroenteritis; Rhinovirus and Influenza A virus lower respiratory tract infection; Staphylococcus coagulase negative bacteremia; ESBL *Escherichia coli* urinary tract infection; Influenza A pneumonitis; Impetigo (methicillin sensitive *Staphylococcus aureus* from wound culture); Staphylococcus epidermis PICC line infection.
Autoimmunity	Likely autoimmune neutropenia, as neutropenia improved with IVIG.
Lymphocytes	Naïve T cells CD45RA^+^↓
Lymphocyte function	Cytokine production ↓; proliferation in response to stimulation ↓
Antibodies	Immunoglobulin level ↑
Myopathy	Congenital muscular hypotonia
Anhidrosis	Anhidrotic ectodermal dysplasia
Other complications	Chronic diarrhea; failure to thrive; small cysts in pancreas; echogenicity in liver and renal cortex.
Outcome	Anti-microbial prophylaxis and immunoglobulin replacement therapy; workup for HSCT.

Because the patient’s symptoms were consistent with primary immunodeficiency (PID), genomic DNA sequencing with a PID gene panel was performed (Invitae) and revealed a homozygous variant of uncertain significance (VUS) in the *ORAI1* gene. This novel missense variant c.376 T > C (p.Cys126Arg) was not found in the Genome Aggregation Database (gnomAD) and the 1000 Genomes Project. In silico analyses by SIFT (0.001), PolyPhen-2 (0.999), and CADD (27.8) predict the variant to be deleterious [24]. The variant was confirmed by Sanger sequencing, which shows that both parents and the healthy sibling are heterozygous (Fig. 1c and S1B). The c.T376C missense mutation affects a highly conserved residue p.C126R in the ORAI1 TM2 domain (Fig. 1d and S2).

### ORAI1 C126R Mutation Abolishes SOCE

To investigate whether C126R impairs CRAC channel function, we measured Ca^2+^ influx in activated peripheral blood mononuclear cells (PBMCs) from the patient, one of the parents, and a healthy control (NC) using the standard Ca^2+^ re-addition assay (Fig. 1e). PBMCs from the parent and healthy control show normal SOCE, whereas in the patient PBMCs SOCE was strongly reduced (62.1 + 1.6%) (Fig. 1e). Inhibition of SOCE could be due to either loss of ORAI1 expression or function. ORAI1 expression was not affected in the patient when assessed at both the protein (Fig. 1f) and mRNA levels (Fig. 1g), showing that the C126R mutation does not affect ORAI1 expression and neither does it alter protein nor mRNA stability.

### ORAI1 C126R Mutation Affects T Cell Proliferation and Cytokine Production

**Fig. 2:**
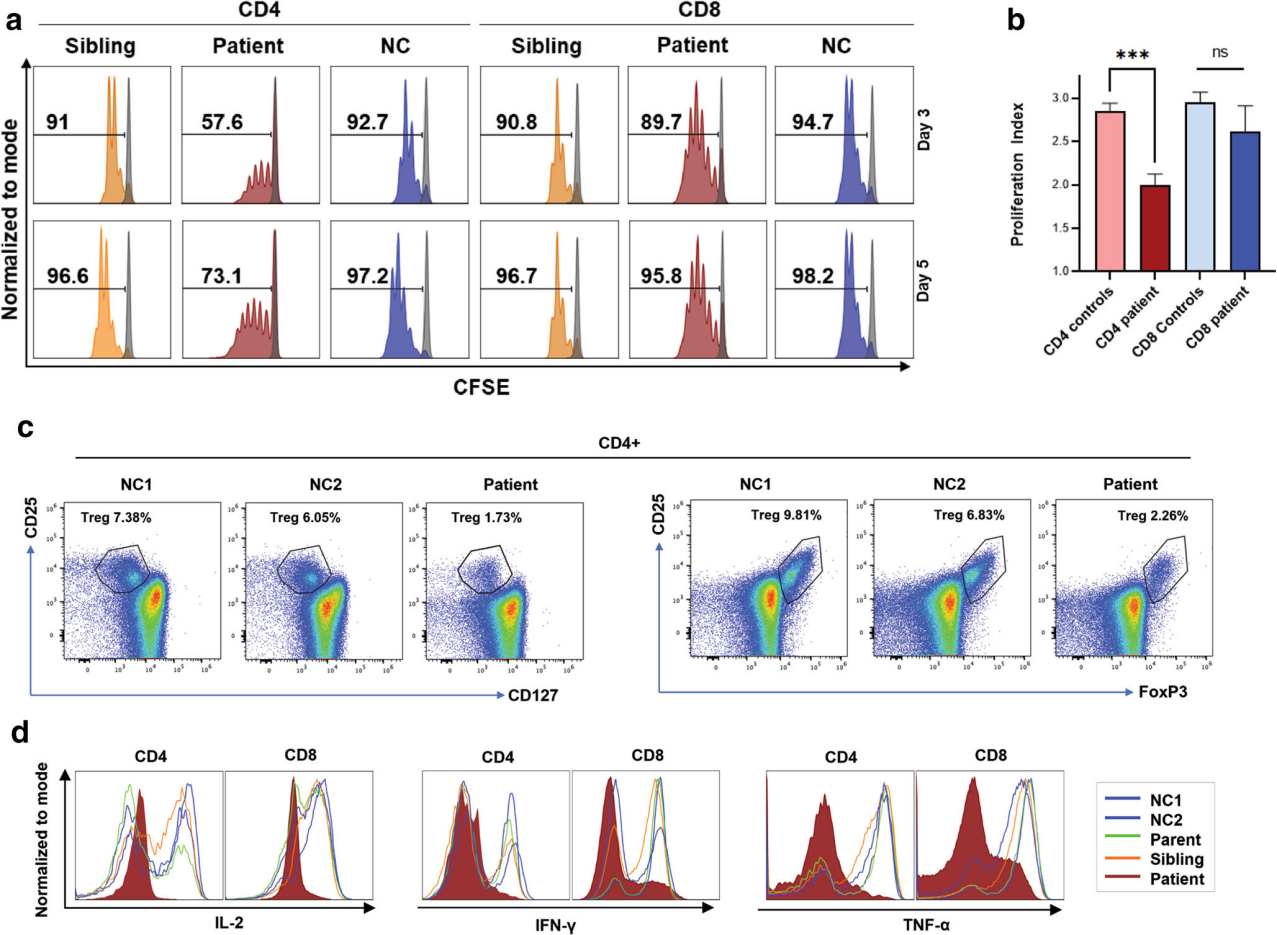
Defective T cell proliferation and function in the patient. **a** Proliferation in PBMCs loaded with CFSE and stimulated with anti-CD2, anti-CD3, and anti-CD28 for 3 or 5 days. Representative CFSE profiles for T cells are shown for days 3 and 5 as indicated. Numbers represent percentage of dividing live cells gated on CD4 and CD8. **b** Proliferation index for day 5 from 3 independent experiments like those presented in panel **a**. For the controls, results from the sibling and two normal controls were pooled. Mean + SEM, *n* = 3 technical replicates, unpaired *t* test, ***p < 0.001. **c** Flow cytometry representative plots of either CD127^low^CD4^+^CD25^+^ or Foxp3^+^CD4^+^CD25^+^ T cells in PBMCs from patient and two healthy donors to assess the percentage of T regulatory cells. **d** Cytokine production by activated T cells from the patient, parent, sibling and healthy control subjects (NC1 and NC2). PBMCs were activated with anti-CD3 and anti-CD28 antibodies for 3 days, then stimulated with phorbol 12-myristate 13-acetate (PMA; 20 ng/mL) and ionomycin (1 μg/mL) for 4 h and analyzed for intracellular cytokine staining using flow cytometry

As SOCE is triggered upon T cell receptor (TCR) engagement and supports cytokine production through NFAT activation, we next investigated the proliferation of patient T cells upon TCR stimulation with anti-CD2, anti-CD3, and anti-CD28 antibodies. Unlike the sibling and normal control, the patient’s CD4^+^ population showed reduced proliferation reflected by reduced percentage of dividing cells in response to TCR stimulation (Fig. 2a). The reduced proliferation of CD4^+^ T cells is statistically significant as assessed by the proliferation index (Fig. 2b). A slight reduction in the dividing CD8^+^ population was observed in the patient (Fig. 2a, b). However, the proliferation index of the CD8^+^ population was not significantly different.

We also assessed the distribution of memory T cells from the patient and controls but did not observe significant alterations in memory T cells subsets (Fig. S3A). We further quantified the percent Treg cells by gating on CD4^+^CD25^+^CD127^low^ cells or CD4^+^CD25^+^Foxp3^+^ cells, and in both cases observe a decrease in the percent of Tregs in the patient as compared to controls (Fig. 2c). This reduction in Foxp3^+^ Tregs has been previously reported in both mice and some human patient with defective SOCE [14, 25, 26]. The reduction in suppressor Treg cells could potentially affect the proliferation of the responding cells in the patient.

To further evaluate the impact on T cell function, we measured cytokine production in activated CD4 and CD8 T cells upon PMA and ionomycin stimulation. Expectedly, production of IL-2, IFN-γ, and TNF-α was greatly reduced in the patient’s T cells in comparison with the parent, sibling, and normal controls (Fig. 2d). Together, these functional assays demonstrate that the patient has impaired T cell proliferation and function.

### The C126R Mutation Impairs ORAI1 PM Localization

**Fig. 3:**
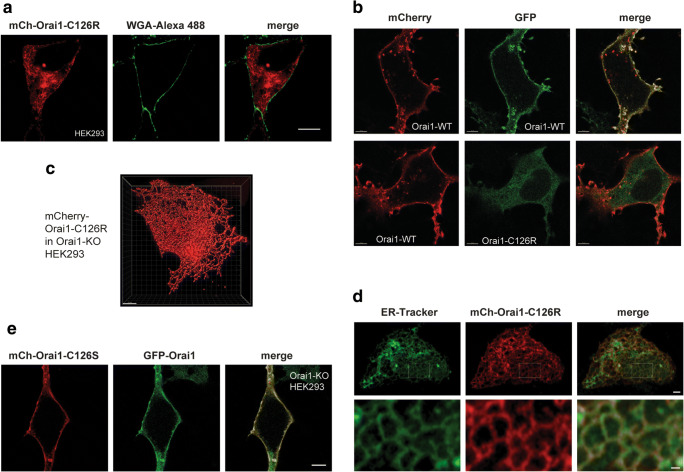
C126R mutation impairs ORAI1 PM localization. **a** Representative confocal airyscan of HEK293 cells expressing mCherry-ORAI1-C126R and co-stained with the PM marker WGA-Alexa 488. Scale bar 10 μm. **b** Confocal images of ORAI1-KO HEK293 cells co-expressing mCherry-ORAI1 (WT) with either GFP-ORAI1 (WT) or GFP-Orai1-C126R as indicated. Scale bar 5 μm. **c** 3D rendering of Orai1-C126R ER localization from a confocal z-stack of images from an Orai1-KO HEK293 cell transiently expressing mCherry-Orai1-C126R using the Imaris software. Scale bar 5 μm. **d** ORAI1-KO HEK293 cells transiently expressing mCh-ORAI1-C126R and stained with ER-tracker (green). Top panel scale bar 2 μm. The area in the white rectangle is magnified in the lower panels to highlight the reticular ER pattern. Scale bar 0.5 μm. **e** Representative confocal images of a cell co-expressing mCherry-ORAI1-C126S with GFP-ORAI1 (WT). Scale bar 5 μm

ORAI1 is the pore-forming subunit of the CRAC channel and localizes to the PM. Because the C126R mutant is expressed at normal levels, we next examined its subcellular localization. Unfortunately, there are no good antibodies to detect endogenous ORAI1 by immunofluorescence. We thus expressed mCherry-tagged ORAI1 C126R in HEK293 cells and co-stained with the PM marker wheat germ agglutinin (WGA). C126R is enriched intracellularly and does not reach the PM (Fig. 3a). In contrast, wild-type Orai1 is enriched at the PM (Fig. 3b). 3D reconstruction shows a reticular intracellular C126R distribution reminiscent of the ER (Fig. 3c). This is indeed the case as confirmed by staining with ER tracker, which colocalizes with ORAI1-C126R (Fig. 3d). Consistent with this finding, when C126R is co-expressed with STIM1 they co-localize to the ER at rest and following store depletion with thapsigargin (Fig. S4). In contrast, wild-type ORAI1 is enriched at the PM at rest and at ER-PM junctions following store depletion (Fig. S4).

The mature ORAI1 channel is a hexamer that could be a heteromultimer of both wild-type and C126R subunits when C126R is overexpressed in HEK293 cells. The fact that mCh-C126R-ORAI1 does not localize to the PM argues that endogenous ORAI1 does not rescue its PM trafficking. However, one caveat with this argument is that transient overexpression results in over tenfold increase in expression over endogenous levels, so the C126R mutant expression could overwhelm endogenous ORAI1. Whether C126R heteromultimarizes with WT ORAI1 is important to understand given that the mutation is recessive and neither the parents nor the sibling (all of whom are heterozygous for C126R) show any defect in SOCE or overt clinical phenotypes.

To directly test whether C126R can be rescued by heteromultimerization with a wild-type subunit, we tagged both WT and C126R with either mCherry or GFP and co-expressed them in different combinations (Fig. 3b). For these experiments to rule out any contribution from endogenous ORAI1, we used a previously characterized ORAI1 knockout (ORAI1-KO) HEK293 cell line generated by CRISPR/Cas9 genome editing (Fig. 3b) [27]. Co-expression of WT ORAI1 tagged with mCherry or GFP shows colocalization of both tags and enrichment at the PM as expected (Fig. 3b, top panels). In contrast, the GFP-tagged C126R mutant localizes intracellularly and is unable to reach the PM even when co-expressed with mCherry-ORAI1 (Fig. 3b, bottom panels). Importantly, we observe no colocalization of the mCh and GFP tags either at the PM or intracellularly, arguing that C126R and WT ORAI1 do not heteromultimerize. This supports a defect in cotranslational membrane insertion of C126R, potentially due to the bulky charged Arg side chain. To test whether the Arg side chain at position 126 is responsible for the folding and trafficking defects of C126R, we replaced the Cys in that position with Ser, which has a smaller polar uncharged side chain. mCherry-C126S when co-expressed with WT ORAI1-GFP traffics normally to the PM and colocalizes with ORAI1 (Fig. 3e).

The phenotype of the heterozygous parents and sibling argue that even partial rescue of the ORAI1 C126R trafficking defect could offer the patient some relief from the clinical symptoms by restoring partial SOCE function. A small-molecule approach would be ideal as it is readily applicable in the clinic. We thus tested whether 4-phenylbutyrate (4-PBA), a chemical chaperone used to relieve ER stress and support protein folding [28], can rescue C126R trafficking. Unfortunately, 4-PBA treatment did not rescue C126R trafficking to the PM (Fig. S3B). We also tested another chemical chaperone glycerol [28] with no success.

### ORAI1 C126R Is Misfolded and Defective in Bilayer Insertion

**Fig. 4:**
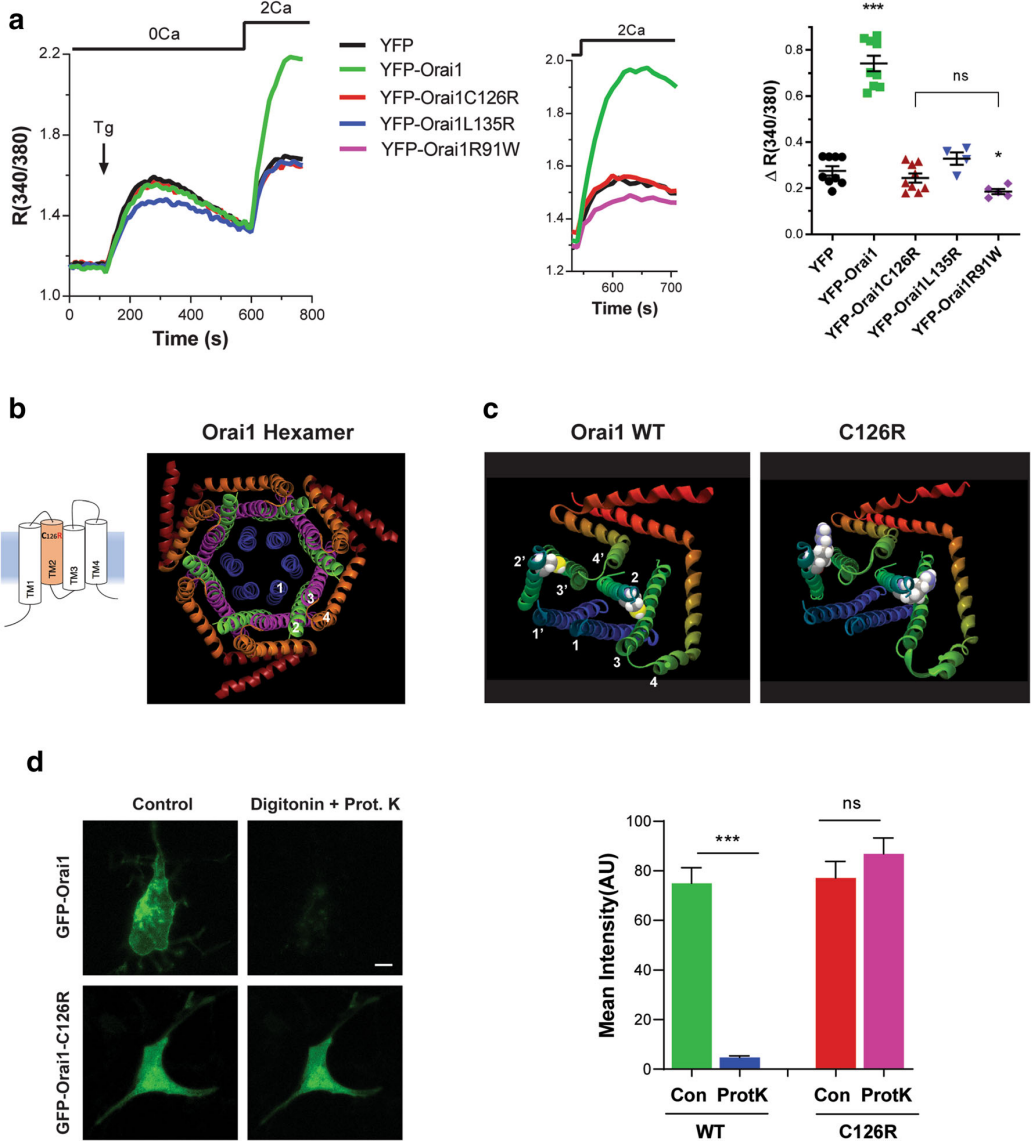
ORAI1 C126R is defective in bilayer insertion. **a** Representative traces and summary data of SOCE responses from ORAI1-KO HEK293 cells expressing either YFP-ORAI1 (WT), YFP-ORAI1-C126R, YFP-ORAI1-L135R, YFP-ORAI1-R91W, or YFP alone. Ca^2+^ stores were depleted with the sarco-endoplasmic reticulum ATPase inhibitor thapsigargin (Tg) in Ca^2+^-free conditions followed by Ca^2+^ re-addition to assess SOCE (*n* = 4–9, mean ± SEM; ***p < 0.0001, *p < 0.05, ns not significant, one-way ANOVA). The stars compare significance to the YFP alone treatment whereas the bracket compares C126R to R91W, which are not significantly different. **b** Cartoon of the ORAI1 monomer and the crystal structure of the *Drosophila* ORAI1 hexamer (4HKR) with individual α-helices numbered. **c** Side view of either wild-type (left panel) or C126R (right panel) human ORAI1 protein structure modeled on the *Drosophila* ORAI1 structure (4HKR). Only two of six ORAI1 subunits are shown for clarity with the Cys and Arg side chains highlighted in a space filling form and the α-helices numbered for each subunit. **d** Example epifluorescence images from the FPP assay for cells expressing either wild-type GFP-ORAI1 or GFP-ORAI1-C126R before (Control) or after digitonin (20 μM) and proteinase K (50 μg/ml) addition, with summary data of mean GFP intensity under the different conditions (*n* = 13, paired *t* test for each group, ***p < 0.0001, ns not significant). Scale bar, 10 μm

The imaging data show that ORAI1 C126R is enriched in the ER and does not traffic to the PM, which should impair function. We confirmed this by quantifying SOCE in knockout ORAI1-HEK293 cells overexpressing the different YFP-tagged mutants or YFP alone as a control (Fig. 4a). Consistent with the imaging data, C126R does not support Ca^2+^ influx above the background observed with YFP alone (Fig. 4a) or as compared to the channel dead mutant R91W (Fig. 4a) [15].

The crystal structure of *Drosophila* ORAI1 reveals a hexameric channel [17]. The ORAI1 pore is lined by six TM1 domains, surrounded by a ring formed of TM2 and TM3, and an outer ring of TM4 and TM4 extensions (Fig. 4b). To gain insights into the effect of the C126R mutation on ORAI1 structure, we modeled both WT human ORAI1 and C126R on the *Drosophila* structure (Fig. 4c). C126 is located toward the extracellular end of the TM2 α-helix. The bulky and positively charged Arg side chain in C126R within the hydrophobic bilayer core is predicted to interfere with transmembrane domains packing (Fig. 4c).

Furthermore, the ER retention of C126R and its inability to colocalize with WT ORAI1 when the two proteins are co-expressed, argue that it is misfolded and does not insert properly into the bilayer. To test this possibility, we utilized the fluorescence protease protection (FPP) assay [23] to assess the topology of C126R in the ER membrane. This approach relies on selective PM permeabilization—which is cholesterol-rich- by digitonin. Membranes of intracellular organelles such as the ER have much lower cholesterol concentrations and are therefore unaffected. Addition of proteinase K degrades fluorescent proteins exposed to the cytoplasm but not the ER lumen. We expressed N-terminal GFP-tagged WT ORAI1 and C126R mutant in ORAI1-KO cells. WT GFP-ORAI1 localizes to the PM with GFP facing the cytosol, resulting in loss of the GFP signal following Proteinase K addition (Fig. 4d). In contrast, the GFP moiety at the N-terminus of C126R is completely protected from proteinase K digestion showing that it localizes to the ER lumen rather than facing the cytosol (Fig. 4d). This argues that C126R is misfolded and does not insert into the bilayer in the correct orientation thus preventing its trafficking to the PM.

**Fig. 5:**
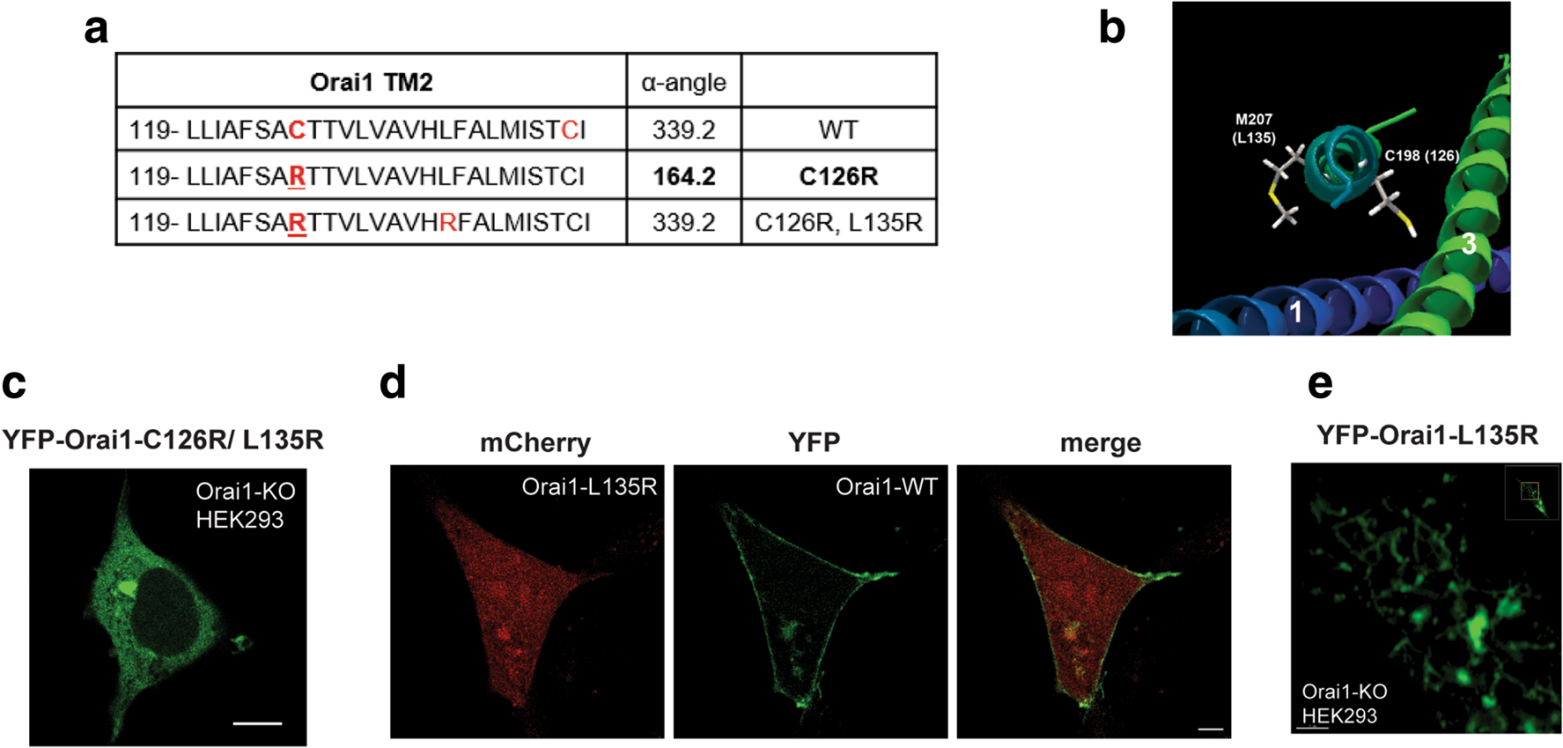
Positively charged Arg side chain in TM2 disrupts ORAI1 folding, membrane insertion, and trafficking. **a** Estimates of the α-helix rotational angle exposed to the lipid bilayer using the Helical Trans-Membrane Segment Rotational Angle (HTMSRAP) algorithm [29] for wild-type ORAI1, the C126R mutant, and the C126R-L135R double mutant. **b** Close up of the ORAI1 TM2 structure showing the side chains for M207 and C198 in the Drosophila structure which correspond to C126 and L135 in the human protein. **c** Confocal image of an ORAI1-KO-HEK293 cell expressing YFP-ORAI1-C126R-L135R double mutant. Scale bar 10 μm. **d** Confocal images of an ORAI1-KO-HEK293 cell expressing mCh-ORAI1-L135R and wild-type YFP-ORAI1 (WT). Scale bar 5 μm. **e** Close-up confocal image of an ORAI1-KO-HEK293 cell expressing ORAI1-L135R to highlight its reticular distribution. Scale bar 2 μm

One possibility for the Arg substitution disrupting ORAI1 membrane insertion is that the charged side chain leads to defective packing and assembly of the ORAI1 hexamer. We therefore assessed the rotational orientation of TM2 using the HTMSRAP algorithm [29], which predicts a decrease in the α-angle of TM2 exposed to the lipid bilayer due to the Arg substitution (Fig. 5a). Interestingly, this is reversed by substituting an Arg at position 135 (L135R), which localizes on the opposite face of TM2 to C126 (Fig. 5a, b). We therefore generated the double mutant C126R, L135R, and assessed its ability to traffic to the PM. The double mutant is retained intracellularly in a similar fashion to C126R (Fig. 5c), showing that the L135R substitution is unable to rescue the C126R trafficking defect.

To test whether the position of the positive Arg side chain on the opposite face of TM2 to C126 is better tolerated for ORAI1 folding, we generated the single ORAI1 L135R substitution and tested its trafficking. Similar to C126R, the L135R mutant is trapped intracellularly even when co-expressed with WT ORAI1 (Fig. 5d) and shows a reticular distribution reminiscent of the ER (Fig. 5e). In addition, the L135R mutant does not support Ca^2+^ influx (Fig. 4a) consistent with its intracellular trapping. These results show that a positive side chain in TM2 within the ORAI1 core is not well tolerated and results in ORAI1 misfolding and trapping intracellularly.

## Discussion

Despite the relatively small number of human cases with STIM1 or ORAI1 mutations, the consistency of clinical presentations due to LoF mutations highlights their critical role in immune function [30]. Here we describe a novel mutation in *ORAI1* that abolishes SOCE and causes CID associated with recurring viral and bacterial infections, neutropenia, muscular hypotonia, and anhidrosis in an infant. This presentation is consistent with the hallmark symptoms of CRAC channelopathy. We show that the patient has largely normal immune cell development, yet T cells are defective in cytokine production and proliferation (Fig. 2a–d). The mutation (*ORAI1* p.C126R) is the first loss of function mutation reported in ORAI1 TM2, and results in defective insertion into the lipid bilayer as shown by the luminal localization of the C126R N-terminus (Fig. 4d). This misfolding leads to ER trapping of C126R and defective trafficking to the PM (Fig. 3). Also, colocalization studies argue that C126R does not heteromultimerize with WT ORAI1 (Fig. 3b). We further show that the defective membrane insertion of C126R is due to the positive Arg side chain within the hydrophobic core of the protein, as the defect is phenocopied by inserting an Arg at position 135 on the opposite face of TM2. Insertion of a positively charged side chain in TM2 is likely to disrupt the tight packing of ORAI1, resulting in misfolding and disrupting trafficking to the PM. Surprisingly though, C126R does not lead to ORAI1 degradation despite its defective bilayer insertion.

Charged residues within the TM domains of integral membrane proteins can be disruptive to protein function, folding, and targeting. For example, insertion of charged residues in the TCR TM domains leads to its ER retention and targeting for degradation [31]. In CFTR, a V232D mutation in TM4 disrupts channel folding and targeting, and leads to protein aggregation when expressed as a peptide [32]. Insertion of arginines into TM peptides from the aspartate receptor disrupts their ability to dimerize and insert into the bilayer [33]. Finally, several human disease-associated mutations of transmembrane proteins frequently include mutations to positively charged residues within the hydrophobic transmembrane core, where they cause severe structural and functional anomalies [34].

However, charged residues within TM domains are not always deleterious and often serve important functions as is the case in voltage-gated channels where basic residues in the fourth TM domain (S4) are critical for sensing voltage changes across the membrane [35]. Acidic residues within the TM domains of the *E. coli* cation-substrate cotransporter [36], and arginines within the TM of the mitochondrial citrate transporter [37], have also been shown to be critical for their transport activity. In addition, charged residues at the TM boundaries have been shown to be important for signaling as shown in GPCRs and rhodopsin [38]. In the case of ORAI1, an arginine at position 91 within TM1 is well tolerated but in this case the positively charged side chain is facing the aqueous pore and not the core of the protein within the bilayer as in the case for C126 [17]. The R91W mutant abolishes SOCE current presumably because the large aromatic tryptophan side chain blocks the channel pore [39, 40].

The ORAI1 C126 residue has been investigated by others previously while generating Cys-less mutants to study the effects of oxidation or pore structure [41, 42]. C126 was mutated to either Ser or Val with no deleterious effects on channel function reported in either study.

Our goal from the detailed cell biological and molecular characterization of the C126R mutant is to explore interventions that may rescue the SOCE defect and the associated immunodeficiency in the patient, thus adopting a personalized medicine approach to better target the treatment. We validate the C126R variant as disease-causing, resulting in directed treatment for immunodeficiencies by providing anti-microbial prophylaxis and immunoglobulin replacement therapy. This treatment regimen has significantly improved the child’s condition, including clearance of infection, normalization of neutrophil numbers (Fig. 1b), resolution of the fever, and improved pulmonary function that no longer requires oxygen supplementation. The child has been discharged after 6 months in hospital. Given that the molecular defect in ORAI1 bilayer insertion and trafficking is unlikely to be resolved by chaperone treatment, the patient has now been worked up for hematopoietic stem cell transplant (HSCT), which has been reported as a curative option for the immunological defects in SOCE-deficient patients [3, 6, 7].

## Supplementary Information

ESM 1 (PDF 1867 kb)

## Abbreviations

SOCE: Store-operated Ca^2+^ entry
GFP: Green fluorescent protein
ER: Endoplasmic reticulum
TM: Transmembrane domain
PM: Plasma membrane
GoF: Gain-of-function
LoF: Loss-of-function
TAM: Tubular aggregate myopathy
CID: Combined immunodeficiency
STIM1: Stromal interaction molecule 1
IP_3_: Inositol 1,4,5 trisphosphate
CRAC: Ca^2+^-release activated Ca^2+^ channel
PID: Primary immunodeficiency
VUS: Variant of uncertain significance
TCR: T cell receptor
NFAT: Nuclear factor of activated T cells
HSCT: Hematopoietic stem cell transplant
PBMC: Peripheral blood mononuclear cell
