# Supplementary material for: Novel ORAI1 Mutation Disrupts Channel Trafficking Resulting in Combined Immunodeficiency
Source: J Clin Immunol. 2021 Mar 1;41(5):1004–15. doi: 10.1007/s10875-021-01004-8 (PMC8249264; doi:10.1007/s10875-021-01004-8)
Supplement: Supplementary file 1 — (PDF 1867 kb) [file 10875_2021_1004_MOESM1_ESM.pdf]

Yu et al.

Supplemental Data

Supplemental Figure 1.

(A) Differential immune cell count and immunoglobulin profile of the patient. (B) Sanger sequence confirmation of the patient showing the homozygous c.376T>C substitution in exon 2 and the heterozygous profile of the parents and sibling compared to the wild type control sequence.

Supplemental Figure 2.

(A) Sequence alignment of Orai1 from different species: human (NP_116179); bovine (NP_001092472); mouse (AAH23149); rat (NP_001014004); *Xenopus* (Q5EAU0); zebrafish (NP_991163) and *Drosophila* (Q9U6B8). The four transmembrane domains are indicated and the conserved Cys at position 126 in the human protein boxed in red. The consensus sequence is shown below the sequences.

Supplemental Figure 3.

(A) Assessment of the different memory T cell populations in two normal controls, the sibling and the patient by gating on CCR7, CD45RA and CD95 as indicated to differentially evaluate the naïve versus central memory (TCM), effector memory CD45RA+ (TEMRA), effector memory (TEM), and stem cell memory T cells. Representative of two technical replicates. No consistent differences in the patient as compared to the controls were observed. (B) Images from an Orai1-KO HEK293 cell transiently co-expressing mCherry-Orai1 (wild-type) and GFP-Orai1-C126R treated with the chemical chaperone 4-PBA (10 μM) 6 hrs following transfection and incubated for 12 hrs before imaging. Images are representative of two independent experiments with multiple dishes. Scale bar 5 μm.

Supplemental Figure 4.

Representative confocal images of Orai1-KO HEK293 cells expressing GFP-STIM1 with either mChery-Orai1 (wild type, ET) or mCherry-Orai1-C126R mutant ar rest (A, rest) or following store depletion with thapsigargin (B, +Tg) as indicated. Scale bar is 10 µm. C126R co-localizes with STIM1 in the ER before and after store depletion. In contrast wild type Orai1 in enriched at the PM at rest and with STIM1 within ER-PM junctions after store depletion.

Figure S1

A

Cell Type	CD Markers	Result	Unit	Normal range
T	$CD3^+$	70 (2630)	% of Lymph (Absolute Count)	39 – 73 (1400 - 8000)
T_h	$CD3^+$ $CD4^+$	55.5 (2082)	% of Lymph (Absolute Count)	25 – 50 (900 - 5500)
T_C	$CD3^+$ $CD8^+$	12 (449)	% of Lymph (Absolute Count)	11 – 32 (400 - 2300)
T_h/T_c	$CD4^+/CD8^+$	4.64		2
B	$CD19^+$	21.2 (794)	% of Lymph (Absolute Count)	17 – 41 (600 - 3100)
NK	$CD3^-$ $/CD16^+/CD56^+$	7.6 (284)	% of Lymph (Absolute Count)	3 – 16 (100 - 1400)
**Immunoglobulins**		**Result**	**Unit**	**Normal range**
	IgG	15.68	g/L	(3.25 – 6.47)
	IgA	2.51	g/L	(0.06 – 0.68)
	IgM	0.71	g/L	(0.00 – 1.3)

B

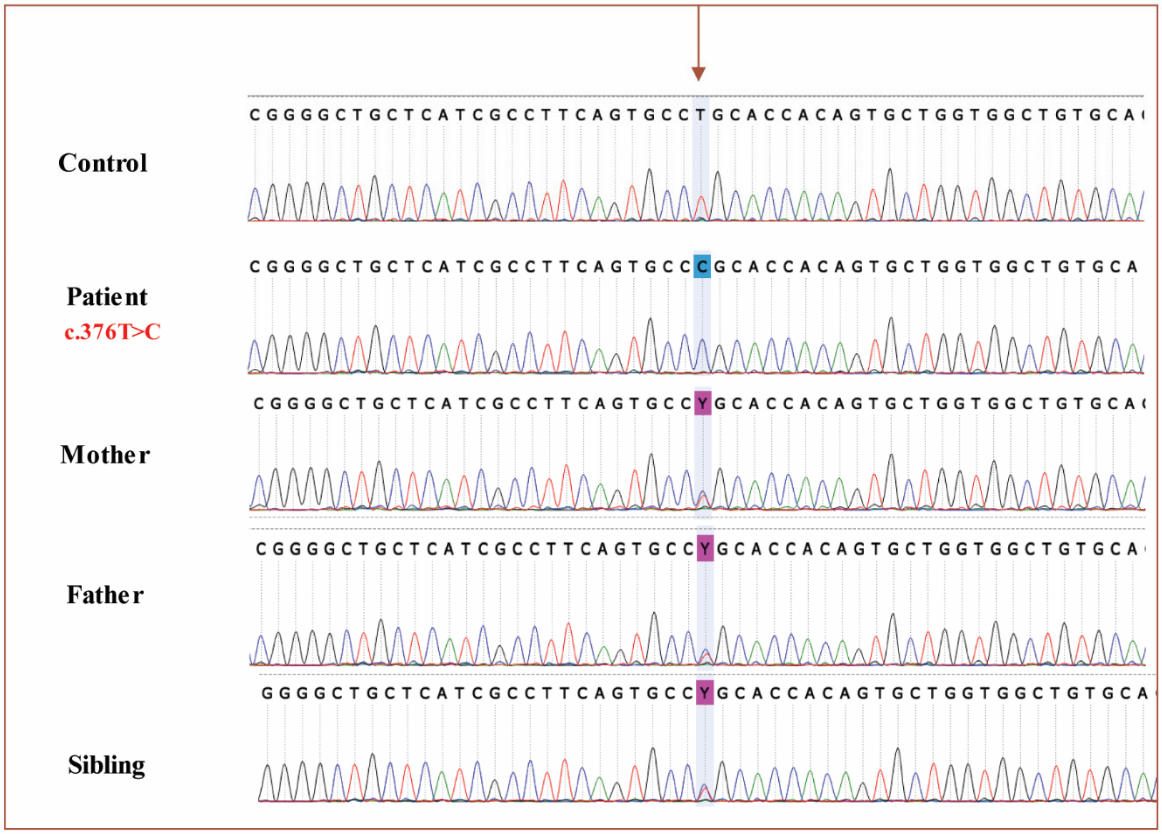

Figure S2

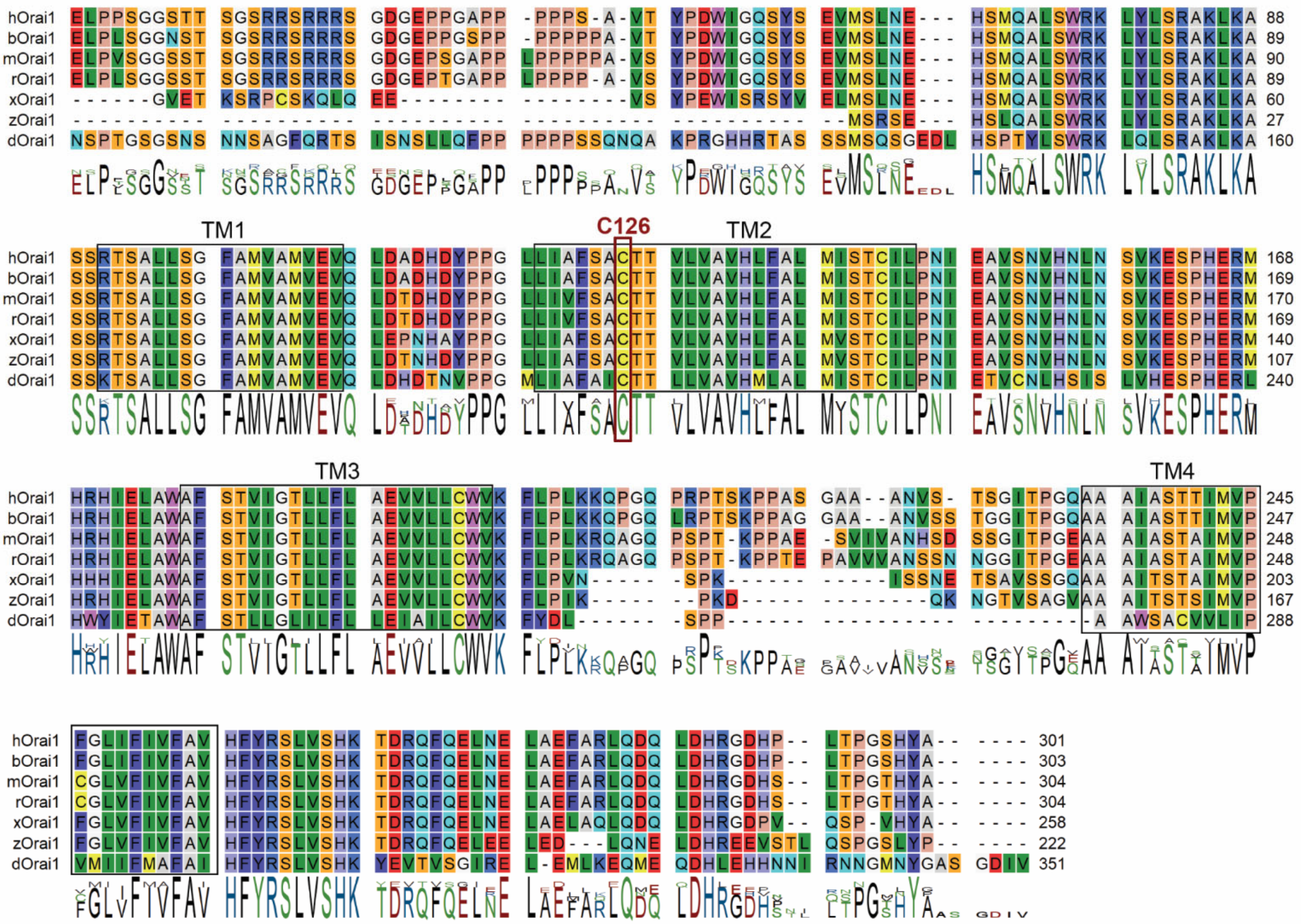

Figure S3

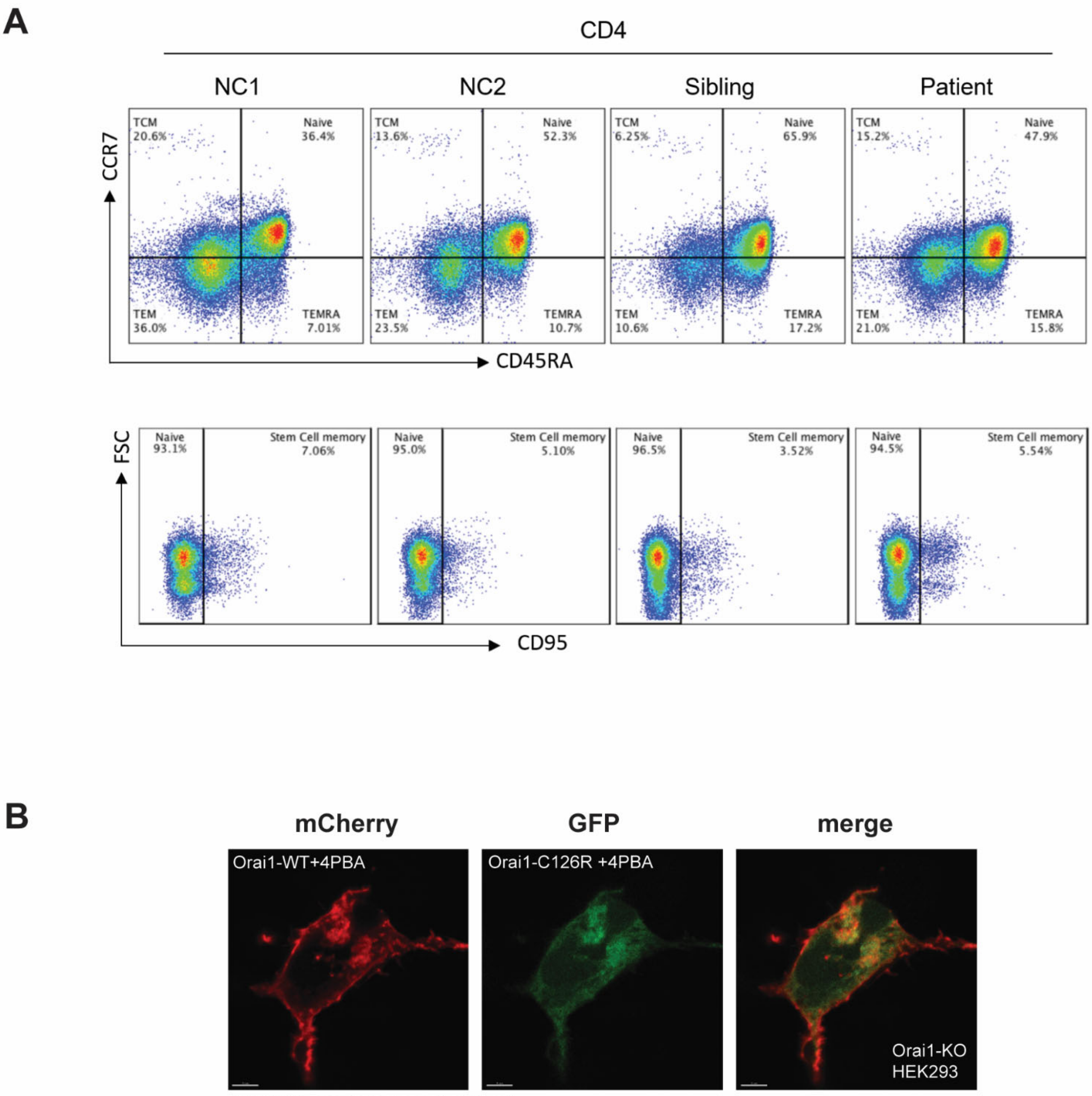

LSM 880, confocol (scale bar, 5um)

Figure S4

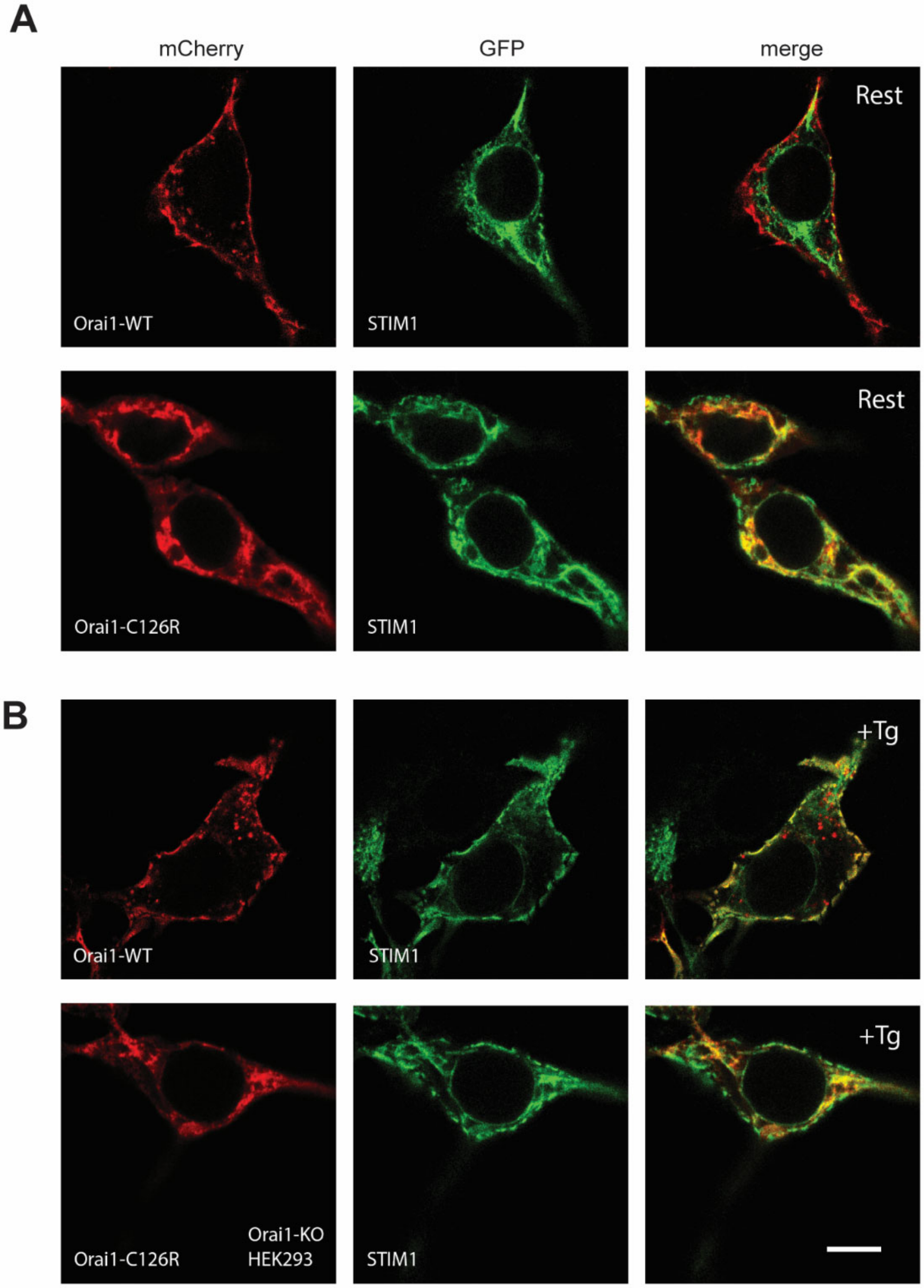

LSM 880, confocol (scale bar, 10um)
